# Data on sediment nitrogen loadings and nitrogen in the biomass of seagrasses from Sungai Pulai estuary (Johor, Malaysia)

**DOI:** 10.1016/j.dib.2019.104979

**Published:** 2019-12-12

**Authors:** Natasha Arina, Che Nurul Ashikin, Nur Hidayah, Mohammad Fairoz, Mohammad Rozaimi

**Affiliations:** Centre for Earth Sciences and Environment, Faculty of Science and Technology, Universiti Kebangsaan Malaysia, 43600 UKM Bangi, Selangor, Malaysia

**Keywords:** Nutrients, Ecosystem service, Anthropogenic influence, Down-core

## Abstract

This paper presents data on nitrogen characteristics in a tropical seagrass meadow located in Sungai Pulai estuary (Johor, Malaysia) and is related to the article “Nitrogen dynamics in tropical seagrass meadows under heavy anthropogenic influence” [1]. Field sampling conducted from August 2015 to May 2016 aimed to collect sediments and seagrass tissues for analysis of nitrogen elemental content and stable isotope values. Sediment samples and seagrass tissue (above-ground and below-ground parts) were collected by using PVC cores. The information is presented as unprocessed and partially data, which incorporates nitrogen content (in %) and δ^15^N values (‰) of sediment and seagrass tissue samples. Nitrogen loadings in the seagrass sediments, as based on down-core data of sediment samples up to 30 cm depths, should be read with [1] to comprehend the baseline nitrogen dynamics of the study area.

**Table:** Specifications Table

Subject	Environmental science
Specific subject area	Marine ecology
Type of data	Table and figure
How data was acquired	Collection and analysis of sediment cores and seagrass tissues. Empirical data was measured in the laboratory by using an elemental analyzer (ECS 4010, Costech Analytical, Valencia CA) and a continuous flow isotope-ratio mass spectrometer (Delta PlusXP, Thermofinnigan, Bremen).
Data format	Raw and partially analysed
Parameters for data collection	Sediment and tissues samples were oven-dried at 60 °C until constant weight was obtained with no further treatment for elemental and isotope analysis.
Description of data collection	Data was collected within the seagrass meadow. Nitrogen elemental content (relative to sediment dry bulk density) and δ^15^N values of species in tissues and sediments are reported.
Data source location	Tanjung Adang shoal (N 01° 19′ 14.3″, E 103° 33′ 54.7″), Merambong shoal (N 01° 20′ 17.5″, E 103° 36′ 11.3″)
Data accessibility	With this article
Related research article	Ashikin, C. N., Rozaimi M., Arina, N., Fairoz, M. & Hidayah, N. Nitrogen dynamics within an estuarine seagrass meadow under heavy anthropogenic influence. Marine Pollution Bulletin. [1], https://doi.org/10.1016/j.marpolbul.2019.110628

**Table:** 

**Value of the Data** • Baseline elemental nitrogen content and stable isotope (δ^15^N) signatures provides insights into nitrogen dynamics in the sampling area • The data provides insights on contemporary nitrogen loadings at local spatial scales within seagrass meadows. • A partial nitrogen budgets based on nitrogen in sediments and seagrass tissues can be constructed by using the data presented.

## 1 Data description

**Fig. 1:**
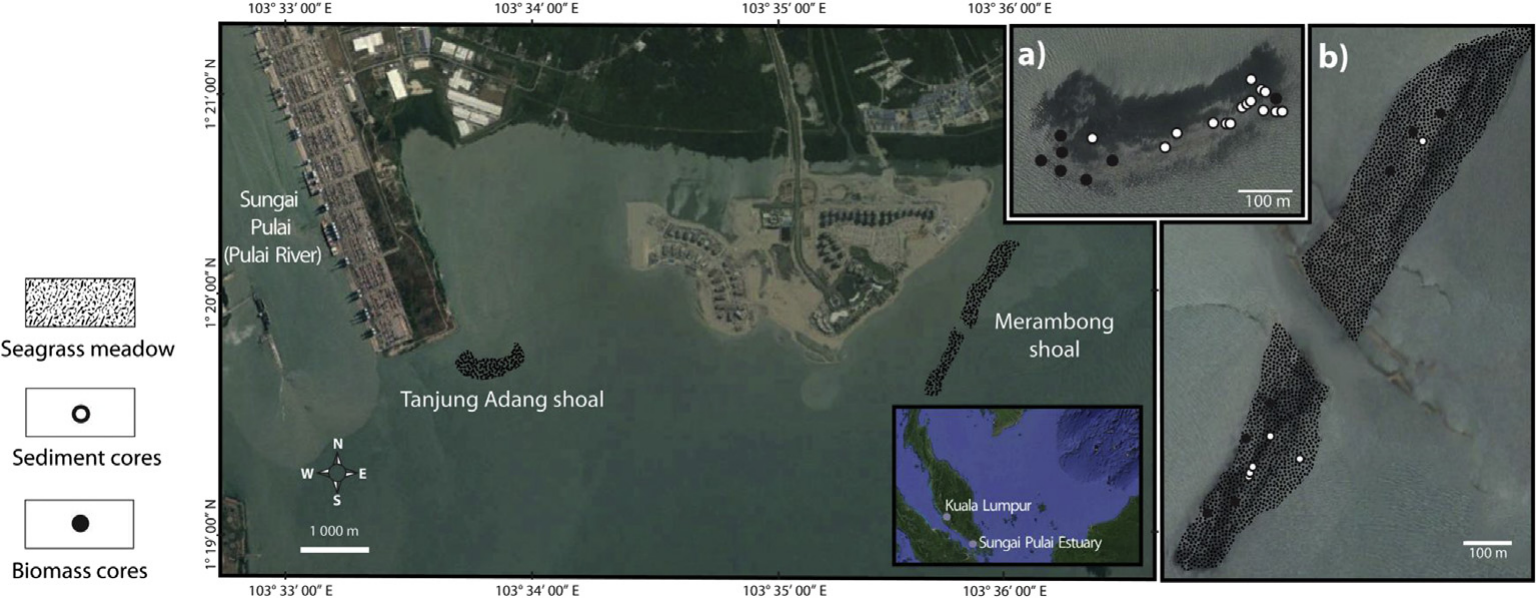
Location of the coring points at the Sungai Pulai estuary seagrass meadows. (a) and (b) are larger-scale maps of Tanjung Adang shoal and Merambong shoal, respectively.

The map of the sampling site is illustrated in Fig. 1. GPS coordinates for sediment and biomass coring points in Tanjung Adang and Merambong shoals are shown in Table 1. Following from the main work in the area [1], sediment nitrogen loadings in the form of cumulative nitrogen N stocks (30 cm sediment depths) and sediment δ^15^N values are presented in Table 2. The information of total biomass (above ground and below ground), and seagrass N nitrogen content in living biomass, is given in Table 3. The sediment down-core profile for %N, and δ^15^N values corresponds to Table 4. Derived C/N ratios for seagrass tissues and sediments are presented in Table 5 and Table 6, respectively.

**Table 1:** GPS coordinates and identification codes of (a) sediment cores; and (b) biomass cores from the sampling location.

Location	Core code	GPS Location
(a)		
Tanjung Adang shoal	1 (Cs)	N1° 19.835′ E103° 34.097′
	2 (Cs)	N1° 19.835′ E103° 34.095′
	3 (Cs)	N1° 19.835′ E103° 34.091′
	4 (Ho)	N1° 19.842′ E103° 34.075′
	5 (Ho)	N1° 19.840′ E103° 34.072′
	6 (Ho)	N1° 19.838′ E103° 34.068′
	7 (Hp)	N1° 19.821′ E103° 34.047′
	8 (Hp)	N1° 19.821′ E103° 34.045′
	9 (Hp)	N1° 19.820′ E103° 34.037′
	10 (Ea)	N1° 19.851′ E103° 34.087′
	11 (Ea)	N1° 19.853′ E103° 34.084′
	12 (Ea)	N1° 19.857′ E103° 34.080′
	13 (Ea)	N1° 19.820′ E103° 33.912′
	14 (Ea)	N1° 19.810′ E103° 33.992′
	15 (Ea)	N1° 19.820′ E103° 33.997′
Merambong shoal	16 (Th)	N1° 19.856′ E103° 35.925′
	17 (Th)	N1° 19.853′ E103° 35.923′
	18 (Th)	N1° 19.851′ E103° 35.922′
	19 (Ea)	N1° 20.292′ E103° 36.188′
	20 (Ea)	N1° 19.868′ E103° 35.990′
	21 (Ea)	N1° 19.885′ E103° 35.955′
(b)		
Tanjung Adang shoal	A1	N1° 19′ 46.6″ E103° 33′ 54.1″
	A2	N1° 19′ 47.3″ E103° 33′ 51.5″
	A3	N1° 19′ 47.4″ E103° 33′ 52.3″
	A4	N1° 19′ 48.3″ E103° 33′ 53.3″
	A5	N1° 19′ 49.2″ E103° 33′ 53.2″
	A6	N1° 19′ 48.6″ E103° 34′ 00.2″
	A7	N1° 19′ 47.6″ E103° 33′ 56.6″
	A8	N1° 19′ 50.9″ E103° 34′ 05.6″
Merambong shoal	M1	N1° 20′ 19.1″ E103° 36′ 12.1″
	M2	N1° 20′ 17.9″ E103° 36′ 11.0″
	M3	N1° 20′ 15.1″ E103° 36′ 08.1″
	M4	N1° 19′ 54.0″ E103° 35′ 57.0″
	M5	N1° 19′ 53.2″ E103° 35′ 55.7″
	M6	N1° 19′ 50.4″ E103° 35′ 54.6″
	M7	N1° 19′ 50.1″ E103° 35′ 53.4″

Abbreviations in parentheses refer to sediment patches designated based on the species’ growth cover: Cs - *Cymodocea serrulata*; Ho - *Halophila ovalis*; Hp - *Halodule pinifolia*; Ea - *Enhalus acoroides*; Th - *Thalassia hemprichii*.

**Table 2:** Nitrogen content by proportion and δ^15^N values of seagrass tissues from Tanjung Adang and Merambong shoals based on species identity.

Sampling location	Species	Part analysed	δ^15^N (‰)	N (%)	N content (g N g^−1^ DW tissue)
Tanjung Adang shoal	*Enhalus acoroides*	Above-ground	8.0	2.74	5.21 × 10^−5^
			7.1	2.61	4.97 × 10^−5^
			9.0	1.87	3.37 × 10^−5^
		Below-ground	5.9	1.23	2.46 × 10^−5^
			5.2	1.10	1.87 × 10^−5^
			4.5	0.88	1.76 × 10^−5^
	*Halophila ovalis*	Above-ground	9.6	1.55	2.48 × 10^−5^
			8.8	1.66	2.99 × 10^−5^
			8.8	1.40	2.38 × 10^−5^
		Below-ground	7.6	0.70	1.32 × 10^−5^
			7.4	1.01	1.51 × 10^−5^
			7.4	0.91	1.46 × 10^−5^
	*Cymodocea serrulata*	Above-ground	7.9	1.21	2.05 × 10^−5^
			5.7	0.90	1.81 × 10^−5^
			6.8	1.06	1.81 × 10^−5^
			8.1	2.35	4.70 × 10^−5^
		Below-ground	9.7	1.88	3.38 × 10^−5^
			9.3	2.12	4.23 × 10^−5^
			7.9	1.96	3.53 × 10^−5^
	*Halodule uninervis*	Above-ground	7.5	2.54	4.07 × 10^−5^
			8.0	1.56	2.95 × 10^−5^
		Below-ground	7.1	0.67	1.33 × 10^−5^
			7.5	0.65	1.11 × 10^−5^
			6.1	0.65	1.18 × 10^−5^
	*Syringodium isoetifolium*	Above-ground	8.9	2.04	3.46 × 10^−5^
			8.8	1.82	3.64 × 10^−5^
			12.0	1.47	2.79 × 10^−5^
		Below-ground	6.6	0.78	1.55 × 10^−5^
			8.2	1.19	2.37 × 10^−5^
			8.5	0.90	1.52 × 10^−5^
Merambong shoal	*Thalassia hemprichii*	Above-ground	9.4	1.51	2.57 × 10^−5^
			11.0	1.06	2.12 × 10^−5^
		Below-ground	10.0	3.19	5.75 × 10^−5^
			11.4	2.47	4.95 × 10^−5^
			10.5	2.95	5.61 × 10^−5^
	*Halodule pinifolia*	Above-ground	8.1	3.38	3.72 × 10^−5^
		Below-ground	8.7	0.74	1.34 × 10^−5^
			7.4	0.55	0.82 × 10^−5^

**Table 3:** Biomass and nitrogen stock values of seagrass species from Tanjung Adang and Merambong shoals.

Location	Species	Core code	Above-ground biomass (g DW m^−2^)	Nitrogen in above- ground biomass (g N m^−2^)	Below-ground biomass (g DW m^−2^)	Nitrogen in below- ground biomass (g N m^−2^)	Total biomass (g DW m^−2^)
Tanjung Adang shoal	*Halodule pinifolia*	A1	5.605	0.190	11.155	0.072	16.760
			0.685	0.023	1.115	0.007	1.800
			1.260	0.043	5.045	0.033	6.305
			0.560	0.019	1.155	0.007	1.715
		A2	2.695	0.091	10.570	0.068	13.265
			2.605	0.088	2.735	0.018	5.340
			4.025	0.136	3.845	0.025	7.870
			0.715	0.024	1.320	0.009	2.035
		A3	5.265	0.178	6.140	0.040	11.405
		A4	2.175	0.074	3.240	0.021	5.415
			4.595	0.155	11.540	0.074	16.135
			0.465	0.016	0.690	0.004	1.155
		A6	0.930	0.031	0.340	0.002	1.270
		A7	1.360	0.046	3.010	0.019	4.370
			0.985	0.033	1.265	0.008	2.250
			1.190	0.040	2.575	0.017	3.765
			3.415	0.116	9.355	0.060	12.770
	*Enhalus acoroides*	A1	23.220	0.559	277.320	2.966	300.540
			34.000	0.819	314.605	3.364	348.605
		A2	443.600	10.688	1915.515	20.484	2359.115
		A4	181.200	4.366	323.905	3.464	505.105
		A5	279.600	6.737	604.200	6.461	883.800
		A6	163.575	3.941	873.550	9.342	1037.125
		A8	28.645	0.690	237.495	2.540	266.140
			77.775	1.874	610.365	6.527	688.140
	*Cymodocea serrulata*	A2	17.360	0.240	77.185	0.153	94.545
			24.435	0.337	60.580	1.203	85.015
			29.820	0.412	38.615	0.767	68.435
			10.040	0.139	19.630	0.390	29.670
		A3	4.925	0.068	10.800	0.214	15.725
		A4	8.335	0.115	39.690	0.788	48.025
			3.580	0.049	14.255	0.283	17.835
			2.020	0.028	3.985	0.079	6.005
		A6	14.570	0.201	13.200	0.262	27.770
			18.960	0.262	24.240	0.481	43.200
			3.260	0.045	32.670	0.649	35.930
			6.485	0.090	37.635	0.747	44.120
		A8	10.990	0.152	31.460	0.625	42.450
			20.785	0.287	68.535	1.361	89.320
			18.800	0.260	77.505	1.539	96.305
			19.660	0.271	61.485	1.221	81.145
			18.445	0.255	30.745	0.610	49.190
	*Halodule uninervis*	A2	7.405	0.049	22.125	0.453	29.530
			0.995	0.007	4.810	0.099	5.805
		A5	5.685	0.037	8.910	0.183	14.595
			3.715	0.024	9.985	0.205	13.700
			3.930	0.026	6.330	0.130	10.260
		A6	2.585	0.017	3.365	0.069	5.950
			2.220	0.015	4.045	0.083	6.265
		A8	2.195	0.014	8.465	0.173	10.660
			1.670	0.011	5.785	0.119	7.455
			5.785	0.038	8.645	0.177	14.430
			9.485	0.062	17.370	0.356	26.855
		A3	3.210	0.021	1.410	0.029	4.620
	*Syringodium isoetifolium*	A8	8.865	0.157	21.180	0.202	30.045
			23.145	0.411	29.620	0.282	52.765
			18.040	0.320	22.735	0.216	40.775
			5.225	0.093	20.540	0.196	25.765
	*Halophila ovalis*	A1	7.800	0.120	6.875	0.060	14.675
			10.330	0.159	13.810	0.120	24.140
			0.900	0.014	1.570	0.014	2.470
			8.155	0.125	8.495	0.074	16.650
			9.470	0.146	10.200	0.089	19.670
		A2	2.440	0.038	1.165	0.010	3.605
			5.575	0.086	2.505	0.022	8.080
			0.750	0.012	1.255	0.011	2.005
		A3	2.430	0.037	12.090	0.105	14.520
			14.820	0.228	16.425	0.143	31.245
			4.860	0.075	1.180	0.010	6.040
			8.880	0.137	9.355	0.082	18.235
			8.320	0.128	11.880	0.104	20.200
		A4	13.955	0.215	19.675	0.172	33.630
			3.525	0.054	2.120	0.018	5.645
			12.525	0.193	8.685	0.076	21.210
			6.905	0.106	7.910	0.069	14.815
		A5	10.625	0.163	4.605	0.040	15.230
			11.095	0.171	17.140	0.149	28.235
			9.975	0.153	6.030	0.053	16.005
			7.165	0.110	13.000	0.113	20.165
			18.290	0.281	23.470	0.205	41.760
		A6	1.560	0.024	2.115	0.018	3.675
			6.080	0.093	7.100	0.062	13.180
			9.810	0.151	8.975	0.078	18.785
			1.760	0.027	1.810	0.016	3.570
			18.750	0.288	16.255	0.142	35.005
		A7	3.435	0.053	3.350	0.029	6.785
			7.580	0.117	13.685	0.119	21.265
			8.720	0.134	13.335	0.116	22.055
			3.930	0.060	5.770	0.050	9.700
			6.155	0.095	7.115	0.062	13.270
		A8	1.695	0.026	2.880	0.025	4.575
			1.190	0.018	0.300	0.003	1.490
			2.715	0.042	1.400	0.012	4.115
			3.670	0.056	5.395	0.047	9.065
			2.350	0.036	3.675	0.032	6.025
Merambong shoal	*Enhalus acoroides*	M2	236.175	5.691	910.535	9.737	1146.710
		M3	46.790	1.127	276.590	2.958	323.380
			126.425	3.046	62.410	0.667	188.835
		M4	158.950	3.830	12.260	0.131	171.210
		M5	162.295	3.910	442.295	4.730	604.590
		M6	161.125	3.882	562.430	6.015	723.555
		M7	26.945	0.649	257.625	2.755	284.570
			100.760	2.428	94.785	1.014	195.545
	*Halodule uninervis*	M1	6.140	0.040	6.680	0.137	12.820
		M4	0.350	0.002	4.580	0.094	4.930
	*Thalassia hemprichii*	M2	15.995	0.206	19.255	0.553	35.250
			5.180	0.067	6.205	0.178	11.385
			6.870	0.088	19.050	0.547	25.920
		M4	28.005	0.360	18.625	0.535	46.630
			9.370	0.120	19.075	0.548	28.445
			48.450	0.623	132.760	3.814	181.210
			31.160	0.401	87.560	2.516	118.720
		M7	1.230	0.016	3.115	0.089	4.345
			13.440	0.173	50.455	1.450	63.895
	*Halodule pinifolia*	M7	6.110	0.207	10.805	0.070	16.915
			4.915	0.166	5.575	0.036	10.490
	*Halophila ovalis*	M1	8.830	0.136	7.750	0.068	16.580
			14.705	0.226	9.260	0.081	23.965
			17.460	0.268	15.030	0.131	32.490
			8.785	0.135	7.075	0.062	15.860
			7.235	0.111	10.075	0.088	17.310
		M2	5.440	0.084	7.675	0.067	13.115
			3.000	0.046	4.750	0.041	7.750
			9.250	0.142	4.985	0.043	14.235
		M3	1.580	0.024	1.235	0.011	2.815
			3.560	0.055	8.305	0.072	11.865
			3.145	0.048	6.025	0.053	9.170
		M4	20.205	0.311	27.000	0.235	47.205
			13.175	0.203	7.055	0.062	20.230
			12.980	0.200	8.815	0.077	21.795
			18.340	0.282	12.710	0.111	31.050
			20.000	0.308	10.430	0.091	30.430
		M5	10.900	0.168	17.645	0.154	28.545
			9.940	0.153	12.060	0.105	22.000
			19.445	0.299	26.760	0.233	46.205
		M6	10.100	0.155	6.870	0.060	16.970
			12.940	0.199	13.415	0.117	26.355
			16.560	0.255	20.035	0.175	36.595
			12.330	0.190	14.625	0.128	26.955
		M7	5.315	0.082	8.795	0.077	14.110
			5.855	0.090	4.670	0.041	10.525
			7.255	0.112	7.080	0.062	14.335
			4.580	0.070	5.295	0.046	9.875

**Table 4:** Downcore data for %N and δ^15^N values of bulk sediments.

Core code	Sediment layer (cm)	N (%)	δ^15^N (‰)	Sediment dry bulk density (g DW cm^−3^)	N stocks (g N m^−2^)
1 (Cs)	0–1	0.021	5.2	2.03	4.2730
	4–5	0.020	3.5	1.66	3.3167
	9–10	0.026	2.8	1.42	3.6886
	14–15	0.029	2.5	1.64	4.7617
	19–20	0.023	2.0	1.67	3.8373
	24–25	0.032	2.0	1.13	3.6135
	29–30	0.054	2.0	0.89	4.8286
2 (Cs)	0–1	0.024	5.0	2.18	5.2319
	4–5	0.019	3.8	1.54	2.9259
	9–10	0.022	3.4	1.53	3.3616
	14–15	0.022	2.2	1.49	3.2716
	19–20	0.033	2.4	1.15	3.7924
	24–25	0.051	2.7	1.18	5.9955
3 (Cs)	0–1	0.021	4.2	2.12	4.4610
	4–5	0.016	3.4	1.53	2.4452
	9–10	0.031	3.5	1.42	4.4173
	14–15	0.023	3.2	1.64	3.7669
	19–20	0.034	2.9	1.04	3.5302
4 (Ho)	4–5	0.027	5.1	1.12	3.0176
	9–10	0.025	7.2	1.73	4.3159
	14–15	0.016	3.5	1.28	2.0407
	19–20	0.030	4.4	0.80	2.3856
	24–25	0.046	2.5	0.95	4.3908
5 (Ho)	0–1	0.017	4.5	1.21	2.0493
	4–5	0.019	4.5	0.94	1.7905
	9–10	0.023	4.1	1.47	3.3757
	14–15	0.014	2.7	1.69	2.3698
	19–20	0.015	3.0	1.23	1.8513
	24–25	0.031	4.0	1.43	4.4198
	29–30	0.034	3.0	1.46	4.9593
6 (Ho)	0–1	0.031	5.1	1.33	4.1149
	4–5	0.029	5.2	1.24	3.5924
	9–10	0.015	3.1	1.70	2.5534
	14–15	0.015	3.3	1.44	2.1632
	19–20	0.024	3.7	1.55	3.7118
	24–25	0.036	2.8	1.72	6.2086
7 (Hp)	0–1	0.012	3.5	1.13	1.3502
	4–5	0.022	4.4	1.29	2.8438
	9–10	0.020	4.4	1.02	2.0401
	14–15	0.014	3.4	1.09	1.5260
	19–20	0.017	5.0	1.47	2.4444
	24–25	0.012	3.5	1.47	1.7611
	29–30	0.015	3.1	1.50	2.2443
8 (Hp)	0–1	0.011	2.5	0.80	0.8812
	4–5	0.012	3.2	1.03	1.2325
	9–10	0.022	4.5	1.65	3.6358
	14–15	0.015	4.5	1.00	1.5098
	19–20	0.012	4.5	1.33	1.5468
	24–25	0.011	2.0	1.24	1.4229
	29–30	0.015	2.0	1.24	1.8142
9 (Hp)	0–1	0.028	2.3	1.41	3.9464
	4–5	0.015	2.2	1.22	1.7787
	9–10	0.013	3.9	1.03	1.3512
	14–15	0.019	4.7	0.83	1.6156
	19–20	0.026	0.7	1.27	3.2831
	24–25	0.033	0.9	0.88	2.8981
10 (Ea)	0–1	0.014	1.3	1.44	2.0643
	4–5	0.014	0.0	1.19	1.6187
	9–10	0.014	3.9	1.36	1.8952
	14–15	0.013	4.7	1.39	1.8781
	19–20	0.042	1.6	1.18	4.9257
11 (Ea)	0–1	0.032	2.6	1.25	4.0400
	4–5	0.022	−0.9	1.28	2.8332
	9–10	0.014	4.7	1.07	1.4724
	14–15	0.018	5.1	1.34	2.3868
12 (Ea)	4–5	0.032	6.0	0.73	2.3143
	14–15	0.032	0.4	0.73	2.3184
	29–30	0.036	0.3	0.98	3.4731
13 (Ea)	0–1	0.010	1.8	1.14	1.0926
	9–10	0.017	3.8	1.30	2.2027
	14–15	0.029	3.0	1.11	3.1779
	19–20	0.026	2.4	1.41	3.7133
	29–30	0.044	2.6	1.43	6.3408
14 (Ea)	0–1	0.031	6.2	0.86	2.6869
	9–10	0.015	5.1	1.29	1.9778
	19–20	0.036	4.8	1.28	4.5753
	29–30	0.030	4.6	1.29	3.8670
15 (Ea)	0–1	0.073	6.5	0.48	3.5030
	19–20	0.040	3.7	0.95	3.7692
	29–30	0.049	3.0	1.10	5.4301
16 (Th)	0–1	0.038	6.5	1.30	4.9072
	4–5	0.024	5.5	1.13	2.7346
	9–10	0.021	2.2	1.62	3.3349
	14–15	0.020	2.6	1.37	2.7550
	19–20	0.021	5.3	1.06	2.2551
	24–25	0.021	4.2	1.09	2.3134
	29–30	0.016	3.9	1.10	1.7273
17 (Th)	0–1	0.020	2.5	1.22	2.4124
	4–5	0.032	2.9	1.39	4.4457
	9–10	0.024	2.3	0.94	2.2572
	14–15	0.023	2.2	1.23	2.8283
	19–20	0.026	2.2	1.51	3.9717
	24–25	0.020	7.0	1.76	3.4716
	29–30	0.024	4.5	0.94	2.2621
18 (Th)	9–10	0.015	3.6	1.39	2.1202
	14–15	0.022	6.9	1.32	2.8597
	19–20	0.028	5.1	1.18	3.2883
	24–25	0.022	4.3	1.05	2.3438
	29–30	0.017	3.5	1.23	2.1024
19 (Ea)	0–1	0.045	9.1	0.70	3.1612
	4–5	0.054	9.7	1.41	7.5504
	9–10	0.021	7.4	1.64	3.4623
	19–20	0.017	6.5	1.52	2.7422
	29–30	0.022	5.7	1.63	3.1917
20 (Ea)	0–1	0.034	8.4	0.77	2.6281
	4–5	0.023	7.7	1.11	2.5937
	9–10	0.022	7.0	1.40	3.1046
	14–15	0.020	6.8	1.78	3.5863
	19–20	0.020	6.3	1.78	3.6308
	29–30	0.019	4.5	1.47	2.7328
21 (Ea)	0–1	0.032	8.4	2.40	7.7570
	9–10	0.036	5.9	1.60	4.9280
	19–20	0.021	6.0	1.67	3.2293
	24–25	0.015	5.9	1.41	2.2531
	29–30	0.024	4.4	1.85	4.5149

Abbreviations in parentheses refer to sediment patches designated as based on the species’ growth cover: Cs - *Cymodocea serrulata*; Ho - *Halophila ovalis*; Hp - *Halodule pinifolia*; Ea - *Enhalus acoroides*; Th - *Thalassia hemprichii.*

**Table 5:** C/N ratio of seagrass species from Sungai Pulai estuary.

Sampling location	Species	Part analysed	C (%)	N (%)	C/N ratio
Tanjung Adang shoal	*Enhalus acoroides*	Above-ground	40.67	2.74	15
			38.56	2.61	15
			38.69	1.87	21
		Below-ground	35.05	1.23	29
			36.05	1.10	33
			34.76	0.88	39
	*Halophila ovalis*	Above-ground	34.85	1.55	22
			34.19	1.66	21
			34.43	1.40	25
		Below-ground	32.51	0.70	47
			34.13	1.01	34
			33.59	0.91	37
	*Cymodocea serrulata*	Above-ground	33.88	1.21	28
			36.53	0.90	40
			35.27	1.06	33
			41.13	2.35	18
		Below-ground	39.76	1.88	21
			39.17	2.12	19
			38.19	1.96	19
	*Halodule uninervis*	Above-ground	42.88	2.54	17
			36.53	1.56	23
		Below-ground	39.68	0.67	59
			39.64	0.65	61
			37.49	0.65	57
	*Syringodium isoetifolium*	Above-ground	38.06	2.04	19
			37.76	1.82	21
			29.22	1.47	20
		Below-ground	34.24	0.78	44
			34.41	1.19	29
			36.95	0.90	41
Merambong shoal	*Thalassia hemprichii*	Above-ground	31.70	1.51	21
			29.05	1.06	27
		Below-ground	39.45	3.19	12
			35.28	2.47	14
			36.79	2.95	12
	*Halodule pinifolia*	Above-ground	44.03	3.38	13
		Below-ground	39.81	0.74	54
			35.80	0.55	65

**Table 6:** Down-core data for C/N ratios of bulk sediment sample.

Core ID	Depth (cm)	C (%)	N (%)	C/N ratio
1 (Cs)	1	0.35	0.021	16
	5	0.38	0.020	19
	10	0.64	0.026	24
	15	0.60	0.029	21
	20	0.38	0.023	16
	25	0.80	0.032	25
	30	1.66	0.054	31
2 (Cs)	1	0.44	0.024	18
	5	0.28	0.019	15
	10	0.42	0.022	19
	15	0.49	0.022	22
	20	0.90	0.033	27
	25	1.59	0.051	31
3 (Cs)	1	0.42	0.021	20
	5	0.25	0.016	16
	10	1.00	0.031	32
	15	0.41	0.023	18
	20	1.06	0.034	31
4 (Ho)	5	0.37	0.027	14
	10	0.50	0.025	20
	15	0.30	0.016	19
	20	0.44	0.030	15
	25	1.41	0.046	31
5 (Ho)	1	0.24	0.017	14
	5	0.36	0.019	19
	10	0.33	0.023	14
	15	0.29	0.014	21
	20	0.25	0.015	17
	25	0.55	0.031	18
	30	1.01	0.034	30
6 (Ho)	1	0.51	0.031	17
	5	0.33	0.029	12
	10	0.22	0.015	14
	15	0.23	0.015	15
	20	0.50	0.024	21
	25	1.15	0.036	32
7 (Hp)	1	0.18	0.012	15
	5	0.42	0.022	19
	10	0.58	0.020	29
	15	0.25	0.014	18
	20	0.29	0.017	18
	25	0.17	0.012	14
	30	0.31	0.015	21
8 (Hp)	1	0.17	0.011	16
	5	0.18	0.012	15
	10	0.39	0.022	18
	15	0.31	0.015	21
	20	0.21	0.012	18
	25	0.33	0.011	28
	30	0.28	0.015	19
9 (Hp)	1	0.55	0.028	20
	5	0.19	0.015	13
	10	0.22	0.013	17
	15	0.45	0.019	23
	20	0.61	0.026	24
	25	0.92	0.033	28
10 (Ea)	1	0.19	0.014	13
	5	0.25	0.014	18
	10	0.18	0.014	13
	15	0.18	0.013	14
	20	1.14	0.042	27
11 (Ea)	1	1.10	0.032	34
	5	0.77	0.022	34
	10	0.18	0.014	13
	15	0.25	0.018	14
12 (Ea)	5	0.31	0.032	10
	15	0.61	0.032	19
	30	0.91	0.036	26
13 (Ea)	1	0.36	0.038	10
	5	0.43	0.024	18
	10	0.30	0.021	15
	15	0.40	0.020	20
	20	0.44	0.021	21
	25	0.37	0.021	17
	30	0.31	0.016	20
14 (Ea)	1	0.27	0.020	14
	5	0.37	0.032	12
	10	0.33	0.024	14
	15	0.37	0.023	16
	20	0.41	0.026	16
	25	0.36	0.020	18
	30	0.44	0.024	18
15 (Ea)	10	0.23	0.015	15
	15	0.36	0.022	17
	20	0.51	0.028	18
	25	0.39	0.022	18
	30	0.35	0.017	21
16 (Th)	1	0.36	0.038	10
	5	0.43	0.024	18
	10	0.30	0.021	15
	15	0.40	0.020	20
	20	0.44	0.021	21
	25	0.37	0.021	17
	30	0.31	0.016	20
17 (Th)	1	0.27	0.020	14
	5	0.37	0.032	12
	10	0.33	0.024	14
	15	0.37	0.023	16
	20	0.41	0.026	16
	25	0.36	0.020	18
	30	0.44	0.024	18
18 (Th)	10	0.23	0.015	15
	15	0.36	0.022	17
	20	0.51	0.028	18
	25	0.39	0.022	18
	30	0.35	0.017	21
19 (Ea)	1	0.35	0.045	8
	5	0.45	0.054	8
	10	0.25	0.021	12
	20	0.27	0.017	16
	30	0.24	0.022	11
20 (Ea)	1	0.31	0.034	9
	5	0.37	0.023	16
	10	0.34	0.022	15
	15	0.25	0.020	12
	20	0.15	0.020	7
	30	0.59	0.019	32
21 (Ea)	1	0.97	0.032	30
	10	0.35	0.036	10
	20	0.22	0.021	10
	25	0.53	0.015	34
	30	0.29	0.024	12

Cs - *Cymodocea serrulata*; Ho - *Halophila ovalis*; Hp - *Halodule pinifolia*; Ea - *Enhalus acoroides*; Th - *Thalassia hemprichii.*

## 2 Experimental design, materials and methods

### 2.1 Sampling site

The sampling activity was conducted in two locales (Tanjung Adang and Merambong shoals) situated within Sungai Pulai estuary off the western side of Johor Straits (Johor, Malaysia, Fig. 1). This estuary is known for the presence of mangrove forests and seagrass meadows [2,3]. Anthropogenic activities that modified the coastal environment had been ongoing since the early 2000s [4,5]. Both sediment and tissue samples were collected in this area for nitrogen analysis.

### 2.2 Coring and biogeochemical processing

Sampling was done from August 2015 until May 2016 when the seagrass bed was exposed from submersion. A total of 21 sediment cores were collected by hammering polyvinyl chloride (PVC) pipes (internal diameter of 50 mm, 50 cm depth) within the sampling area. The distal end of the core barrel was modified into a hypodermic design [6] that helped the intrusion into the sediment bed. Sediment cores from Tanjung Adang shoal were sampled from *Cymodocea serrulata*, *Halophila ovalis, Enhalus acoroides* and *Halodule pinifolia* sediment patches. In Merambong shoal, samples were collected from *Enhalus acoroides* and *Thalassia hemprichii* sediments. Tissues samples were collected from pre-allocated sampling points by using a biomass corer (internal area 0.02 m^2^, 15 cm depth). The biomass corer was pushed into the sediment surface and whole shoots found within the internal cavity of the biomass corer were collected. Seagrass samples were separated according to species and plant parts (i.e. above-ground versus below-ground tissues). After the plant parts were cleansed off extraneous particles and epiphytes with distilled water, samples were oven-dried (60 °C) until constant weight was attained. Biomass values were measured by using the formula:

biomass (g DW m^2^) = dry weight (g) x internal area (m^2^) of corer base

Sediment cores brought back to the laboratory were extruded from the core barrel and sliced in 1 cm layers. The sub-sampled layers were oven-dried (60 °C) to get constant dry weight. Tissue samples were taken from *C. serrulata*, *H. ovalis, E. acoroides, H. pinifolia*, *H. uninervis* and *Syringodium isoetifolium* above-and ground parts, cleaned as per the biomass-processing step above, and dried. Then, the sediment and tissue samples were ground into fine particles by using a ball-mill grinder. The powdered sediment (70–120 mg) and seagrass tissue samples (1–2 mg) were enclosed in tin capsule for elemental nitrogen and δ^15^N analysis in a continuous flow isotope ratio mass spectrometer (IRMS) analyser (Delta plus XP) at the Stable Isotope Core Lab in Washington State University. δ^15^N values were referenced to atmospheric nitrogen as the standard and reported as (in ‰).

Nitrogen contents were reported as the percentage (%N) of bulk sediments, or tissue dry weight. Sediment %N values were multiplied by the dry bulk density of sediment (g DW cm^−3^) to obtain its nitrogen density (g N cm^−3^). The nitrogen density values were multiplied by 0.3 m (i.e. the length of the sediment core) to obtain the nitrogen loadings as cumulative nitrogen mass (g N m^−2^) in sediments up to 30 cm depths. The percentage nitrogen (%N) was multiplied by the sample encapsulated weights (g) to obtain the proportion of nitrogen content within dried seagrass tissues (as g N g^−1^ DW tissue). Nitrogen content (%N), as per species identity, was multiplied by biomass values (above- and below-ground, in g DW m^−2^) of that species to obtain the nitrogen stocks (g N m^−2^) in seagrass tissues.

## References

[1] Ashikin C.N., Rozaimi M., Arina N., Fairoz M., Hidayah N. (2019). Nitrogen dynamics in tropical seagrass meadows under heavy anthropogenic influence. Mar. Pollut. Bull..

[2] Kamarrudin I., Mohamed C.A.R., Rozaimi M., Alfian B.A.A.K., Fitra A.Z., Lee J.N. (2011). Malaysia's Marine Biodiversity: Inventory and Current Status.

[3] Juliana W.A.W., Razali M.S., Latiff A. (2014). Distribution and rarity of Rhizophoraceae in Peninsular Malaysia. Mangrove Ecosystems of Asia. Status, Challenges and Management Strategies.

[4] Japar Sidik B., Muta Harah Z., Short F.T., Finlayson C.M., Milton G.R., Prentice R.C., Davidson N.C. (2016). Seagrass in Malaysia: Issues and challenges ahead. The Wetland Book: II: Distribution, Description and Conservation.

[5] Shi G.W., Ghaffar M.A., Ali M.M., Cob Z.C. (2014). The Polychaeta (Annelida) communities of the Merambong and Tanjung Adang shoals, Malaysia, and its relationship with the environmental variables. Malay. Nat. J..

[6] Rozaimi M., Fairoz M., Hakimi T.M., Hamdan N.H., Omar R., Ali M.M., Tahirin S.A. (2017). Carbon stores from a tropical seagrass meadow in the midst of anthropogenic disturbance. Mar. Pollut. Bull..

